# Constraints and prospects for contraceptive service provision to young people in Uganda: providers' perspectives

**DOI:** 10.1186/1472-6963-11-220

**Published:** 2011-09-17

**Authors:** Gorrette Nalwadda, Florence Mirembe, Nazarius M Tumwesigye, Josaphat Byamugisha, Elisabeth Faxelid

**Affiliations:** 1 Department of Nursing, School of Health Sciences, Makerere University College of Health Sciences, P.O Box 7072, Kampala, Uganda; 2 Department of Obstetrics and Gynecology, School of Medicine, Makerere University College of Health Sciences, P.O Box 7072, Kampala, Uganda; 3 Department of Epidemiology and Biostatistics, School of Public Health, Makerere University College Health Sciences, P.O Box 7072, Kampala, Uganda; 4 Division of Global Health (IHCAR), Department of Public Health Science, Karolinska Institutet, Sweden

## Abstract

**Background:**

Unintended pregnancies lead to unsafe abortions, which are a leading cause of preventable maternal mortality among young women in Uganda. There is a discrepancy between the desire to prevent pregnancy and actual contraceptive use. Health care providers' perspectives on factors influencing contraceptive use and service provision to young people aged 15-24 in two rural districts in Uganda were explored.

**Methods:**

Semi-structured questionnaires were used for face- to-face interviews with 102 providers of contraceptive service at public, private not-for-profit, and private for-profit health facilities in two rural districts in Uganda. Descriptive and inferential statistics were used in the analysis of data.

**Results:**

Providers identified service delivery, provider-focused, structural, and client-specific factors that influence contraceptive use among young people. Contraceptive use and provision to young people were constrained by sporadic contraceptive stocks, poor service organization, and the limited number of trained personnel, high costs, and unfriendly service. Most providers were not competent enough to provide long-acting methods. There were significant differences in providers' self-rated competence by facility type; private for-profit providers' competence was limited for most contraceptives. Providers had misconceptions about contraceptives, they had negative attitudes towards the provision of contraceptives to young people, and they imposed non-evidence-based age restrictions and consent requirements. Thus, most providers were not prepared or were hesitant to give young people contraceptives. Short-acting methods were, however, considered acceptable for young married women and those with children.

**Conclusion:**

Provider, client, and health system factors restricted contraceptive provision and use for young people. Their contraceptive use prospects are dependent on provider behavior and health system improvements.

## Background

High fertility is associated with preventable maternal and infant morbidity and mortality in low-resource settings with weak health care infrastructure [1]. Research indicates that contraceptive use alone could reduce maternal deaths associated with unwanted pregnancies by 40 percent [2]. Contraceptive use in sub-Saharan Africa is low at only 21 percent, mainly as a result of inaccessibility to fertility regulation methods [3,4]. The proportion of satisfied demand for contraception is lower, especially among young people [5].

Uganda's reproductive health indicators continue to be poor, with a maternal mortality ratio of 435/100,000 live births. The total fertility rate is 6.7 children per woman, and teenage pregnancies constitute 25 percent of all pregnancies [6]. Birth intervals remain short, and Ugandan women have more than three children by their late 20s [6]. Nearly half of the 1.4 million annual pregnancies occurring in Uganda are unwanted [7]. Unintended pregnancies have been linked to unsafe abortions that constitute nearly one third of maternal deaths among young people in Uganda [8,9].

The use of modern contraception is extremely limited in Uganda. Only 17 percent of all women of reproductive age, and 18 percent of married women, utilize modern contraception [6,7]. Uganda lags behind in comparison with other countries in the region such as Kenya, Rwanda, and Tanzania, where 39, 27, and 20 percent of married women in each country, respectively, use modern contraceptive methods [10]. The paradox is that two in every five women in Uganda want to space or limit childbirth, but are not using contraceptives [7]. The unmet need for contraceptive use is therefore 2-3 times higher than the current use of contraceptives, and women exceed their desired fertility by two children [6,7]. Despite a liberal family planning policy that allows access to contraceptive services to every sexually active individual and couples irrespective of age [11], a large proportion of sexually active Ugandan young people have never used contraceptives. The policy guidelines target everyone in need of contraception and young people is one group which is prioritized. Noteworthy, contraceptives are free in public facilities and private facilities charge low fees as a commercial marketing strategy. While awareness of at least one contraceptive method is as high as 98 percent [6], only 10-20 percent of young people report ever using modern contraceptives apart from condoms [12]. Detailed knowledge about how to prevent an unwanted pregnancy is, however, low among young people [13]. The apparent awareness has not transformed into contraceptive use, and the reasons for this are unclear. Therefore, young people remain at risk of unintended pregnancy and unsafe abortion with subsequent mortality and morbidity.

A randomized, controlled trial in Uganda reported high levels of unwanted pregnancy in a setting where contraceptives were made available, warranting further investigation to better understand why contraceptives were not used [14]. In one recent focus group discussion study, young people cited distance, provider attitudes, and unfriendly service as barriers to contraceptive use [15]. Another previous study noted that provision of sexual and reproductive health information and service for young people is weak [5]. Medical, economic, cultural and social barriers as well as moral opinions, and religious beliefs that constrain access have been highlighted in literature [16]. There are, however, gaps in existing literature on providers' perspectives, and studies have recommended research to better understand providers' views on access to contraceptive services for young people [17,18]. Research has neither identified nor quantified providers' practices that encourage or help clients to achieve their desired family size [19]. It is unclear how providers' stances influence the services young people receive. Addressing barriers to the use of contraceptive services requires detailed and context-specific information, which is often lacking [20]. Furthermore, health care system factors that might influence contraceptive use among young people are not well-understood. The aim of this study was, therefore, to explore providers' perspectives on factors influencing contraceptive use and service provision to young people aged 15-24 in two rural districts in Uganda. Identifying such factors could inform policy and suggest context-specific strategies to improve contraceptive service provision for young people in order to reduce unintended pregnancy.

## Methods

### Study site and population

A cross-sectional study was conducted between August, 2008 and February, 2009 in the Mityana and Mubende districts in Uganda. In 2008, the populations in the two districts was projected to be 333,300 and 539,700 for Mityana and Mubende, respectively; both at an annual population growth rate of 3.6 percent [21]. More than 60 percent of the population in the two districts was estimated to be less than 18 years old [22]. Based on the demographic health survey regional data, both districts had teenage pregnancy rates of 30 percent compared to 25 percent nationally and a total fertility rate of nearly eight children per woman [6]. Each district has three health sub-districts (HSD). Each HSD covers a catchment population of approximately 500,000 people. The HSD with a hospital was purposively selected as the study site to ensure representation of all types of health facilities. Three types of health facilities exist: government (public), private not-for-profit (PNFP), and private for-profit (PFP). The study population was health care professionals who provided contraceptive services. Health care professionals at all health facilities as well as drug stores providing contraceptives were eligible for the study. Hospitals and health centers constituted the public and PNFP health facilities, while PFP facilities included private clinics, pharmacies, and drug stores.

### Data collection

Public and PNFP health facilities were identified using recent Ministry of Health lists from the two district health offices. Similarly, PFP facilities were identified using district lists of pharmacies and drug stores from the National Drug Authority compiled in 2008. The list of private clinics in the two districts was obtained from the available registry of 2005. The lists consisted of 35 public (2 hospitals, 33 health centers), 11 PNFP (all health centers), and 84 PFP health facilities (20 clinics, 4 pharmacies, 60 drug shops). The inclusion criterion was that health facilities and drug stores should offer any contraceptive method including fertility awareness methods during the study period, 30 drug stores that did not offer contraceptives were excluded. The health care provider in charge of maternal health/family planning services from each of the health centers and private clinics was selected and interviewed. In each of the two hospitals, two providers, one from the family planning unit and another from the maternity unit, were selected and interviewed. This was done because both units provided contraceptive service and information. While in drug stores and pharmacies, the most conversant person in provision or selling of contraceptives was identified and interviewed. All facilities had at least a representative interviewed, and participated in the study.

A semi-structured questionnaire was used to guide face- to-face interviews with 102 health care providers from 100 service delivery points. The interviews were conducted in English at the respective health facility, and each interview lasted 45 to 60 minutes. The questionnaire consisted of open and close-ended questions. Open-ended questions were used to provide greater depth on experiences, views, and attitudes of the providers [23]. The questionnaire was designed to investigate providers' attitudes, behavior, and skills; their views about health care system obstacles and factors leading to contraceptive use; and providers' preparedness to provide contraceptive services to young people. The questionnaire was pre-tested and changes made before data collection started. Providers were also asked to rate their own competencies in three areas: comprehensive counseling for young people, their ability to provide various contraceptive methods, and management of side effects related to contraceptives. Providers rated their competency to provide contraceptive services on a scale of 0-3 as "don't have skills" (0), "require a lot of practice" (1), "require some practice" (2), or "fully competent" (3).

After the interviews, a brief health facility audit of contraceptive services was carried out in all the selected facilities using a checklist for physical infrastructure for privacy, basic contraceptive equipment, commodities in stock, supplies, information, education and communication materials, and record keeping. The aim of the audit was to find out the basic amenities available related to provision of contraceptives. The first author (GN) conducted some of the interviews and facility assessments, and also supervised the research assistants during the remainder of the data collection process. The research assistants were nurse/midwives with field research experience who were trained for two days on study procedures and data collection. The research assistants also participated in the pretesting of the questionnaire and making revisions before use.

### Data management and analysis

Responses from the open-ended questions were coded by the first author. The codes were discussed and agreed upon with the co-authors. Data from the closed questions and the coded data from the open-ended questions were entered using EpiData v3.1 and then cleaned and exported to SPSS V.16 for analysis. The data were analyzed to show characteristics of the providers, availability of contraceptive methods and education materials, and also providers' self-assessment of their competence and consent requirement for contraceptive service provision by facility type. The mean self-assessment score was computed for each of contraceptive service offered and Analysis of Variance (ANOVA) was carried out to test the significance of the difference in the means across the types of health facilities. The assessment score is not continuous but it tended to normality hence making ANOVA a suitable method of analysis. Chi-square test was used to assess the significance of the difference in provider's background characteristics, availability of contraceptive methods at health care facilities, and consent requirements by facility type. The level of statistical significance was set at 0.05.

### Ethical considerations

The higher degrees, research and ethics committee of the Faculty of Medicine, Makerere University and the Uganda National Council for Science and Technology approved the study. Written consent was secured from all participants after explaining the purpose of the study. Participation in the study was voluntary and confidentiality was assured.

## Results

### Characteristics of contraceptive service providers

Overall, most of the facilities were PFP (54%), followed by public facilities (35%), and PNFP facilities were fewer (11%). Two thirds of the study participants were nurses and midwives (67%). However, more than a fifth of the providers were nursing assistants with limited medical training. A higher proportion of the providers in the public health facilities 24 (65%) had received training in family planning compared to those in PNFP 3(27%) and PFP 12 (22%) health facilities (p < 0.001). The median years of experience related to family planning service was six (Table 1). Notably, most training had been completed more than six months before the survey time (69%). Many providers (74%) had recently provided contraceptives to young people.

**Table 1:** Characteristics of providers by facility type

	Health facility type				
	
Provider Characteristics	*Total* *n = 102^*	*Public* n = 37	*PNFP* n = 11	*PFP* n = 54	*Chi-sq* *p-value*
*Professional discipline *					
Medical officer	6 (5.9)	0 (0.0)	0 (0.0)	6 (11.1)	
Clinical officer	6 (5.9)	1 (2.7)	1 (9.1)	4 (7.4)	*
Midwive/nurse	68 (66.7)	30 (81.1)	9 (81.8)	29 (53.7)	
Nursing assistant/others	22 (21.6)	6 (16.2)	1 (9.1)	15 (27.8)	
*Family planning training*					
Yes	39 (38.2)	24 (64.9)	3 (27.3)	12 (22.2)	
No	63 (61.8)	13 (35.1)	8 (72.7)	42 (77.8)	***
*Years of experience in FP provision*					
= < 5	50 (49.0)	13 (35.1)	5 (45.5)	32 (59.3)	
6-10	32 (31.4)	15 (40.5)	4 (36.4)	13 (24.1)	*
>*1*0	20 (19.6)	9 (24.3)	2 (18.2)	9 (16.7)	

^Two providers were interviewed from each hospital *p < 0.05; **p < 0.01; ***p < 0.001; Percentages within brackets. Note that the number of responses in some cells is too small, FP-Family planning, PNFP- Private not for profit, PFP- private for profit

### Contraceptive methods provided and educational materials available

Most of the public and PFP health facilities provided oral pills, progestin-only injections (DMPA), and male condoms. However, availability of the methods differed significantly by facility type (p < 0.001), with PNFP health facilities providing the fewest methods. Long-acting methods such as the Copper Intra Uterine Device (IUD) (6%), implants (6%), and tubal ligation and vasectomy (5%) were the least-provided methods in all health care facilities. Fertility awareness methods (FAM) including rhythm, moon beads, and periodic abstinence were predominantly provided by PNFP (54%, n = 11), (Table 2). Surprisingly, these facilities had no signs, posters, or educational materials to promote FAM. Other methods such as female condoms, combined patches, vaginal rings, cervical caps, spermicides, diaphragms, one or two monthly injections, and levonorgestrel IUDs were not available in any of the facilities. None of the pharmacies or drug stores sold IUDs, implants, or barrier methods other than condoms.

**Table 2:** Contraceptive methods and educational materials by facility type

	Health facility type				
	
Contraceptive methods/educational materials available	*Total* *n = 102*	*Public* *n = 37*	*PNFP* *n = 11*	*PFP* *n = 54*	*Chi-sq* *p-value *
*Contraceptive methods available*					
Progestin only injection	86 (84.3)	35(94.6)	5(45.5)	46(85.2)	***
Male condom	86 (84.3)	33(89.2)	6(54.5)	47(87.0)	**
Oral pills	82 (80.4)	33(89.2)	4(36.4)	45 (83.4)	***
FAM(rhythm, beads, periodic abstinence)	11(10.8)	1(2.7)	6(54.5)	4(7.4)	***
Implants (implanon and norplants)	6 (5.9)	5(13.5)	1(9.1)	0 (0.0)	**
Copper-IUD	6 (5.9)	4(10.8)	1(9.1)	1(1.9)	NS
Vasectomy/Tubal ligation	5(4.9)	4(10.8)	0 (0.0)	1(1.9)	NS
*FP education materials available*					
Family planning IEC materials	38 (37.3)	19 (51.4)	4 (36.4)	15 (27.8)	***
RH/FP guidelines/policy	11 (10.8)	8(21.8)	1 (9.1)	2(3.7)	NS
None	53 (52.0)	10 (27.0)	6(54.5)	37 (68.5)	NS

*p < 0.05; **p < 0.01; ***p < 0.001, NS-Not significant, Percentages within brackets, Note:number of respondents too small in some cells. IEC-Information Education Communication, RH- Reproductive health, FP- Family planning, FAM- fertility awareness method. PNFP- Private not for profit, PFP- private for profit

Guidelines and national policy on reproductive health and family planning were only available in 11 of the 102 facilities (10.8%). Availability of educational materials differed by facility type (p < 0.001), with lower proportion of PFP facilities having educational materials. In addition, family planning signposts were available at all public and PNFP health facilities (Table 2). However, only 12 percent of the health facilities and drug stores provided at least five contraceptive methods.

### Provider competence/skills in offering contraceptive services

Overall, providers considered themselves highly competent (score range 0-3) to provide pills, progestin-only injections, and condoms. However, on average, providers did not feel competent enough to provide IUDs ($\bar{\text{x}}$ = 0.39), implants ($\bar{\text{x}}$ = 0.47), emergency contraceptives ($\bar{\text{x}}$ = 1.05), and barrier methods other than condoms ($\bar{\text{x}}$ = 0.42). With the exception of FAM and IUD insertion, mean competence scores were higher among providers in public facilities compared to those in PNFP and PFP facilities. Providers in PNFP health facilities rated their competence regarding providing FAM to be higher compared to providers in public and PFP facilities (p < 0.01). Providers from public, PNFP, and PFP facilities rated themselves nearly equally low on competence in providing emergency contraceptives ($\bar{\text{x}}$ ≈ 1), IUD ($\bar{\text{x}}$ < 0.65), implants ($\bar{\text{x}}$ ≈ 0.46), and barrier methods (diaphragms, vaginal rings, cervical caps, spermicides) ($\bar{\text{x}}$ < 0.80). Providers' competences differed significantly by health facility type with respect to comprehensive counseling (p < 0.001), FAM (p < 0.01), injections (p < 0.05), barrier methods (p < 0.05), and management of side effects (p < 0.05) (Table 3).

**Table 3:** Providers' self-assessment score of competence to offer contraceptive services

	Health facility type					
	
Contraceptive Services	*Total* *(n = 102)* *Mean (SD)*	*Public* *(n = 37)* *Mean (SD)*	*PNFP* *(n = 11)* *Mean (SD)*	*PFP* *(n = 54)* *Mean (SD)*	*ANOVA* *F-value*	*p- value*
Comprehensive counseling	1.68 (0.9)	2.1 1(0.57)	1.73 (0.47)	1.37(1.0)	8.94	***
IUD Insertion	0.39 (0.86)	0.27 (0.77)	0.64 (1.12)	0.43 (0.86)	0.86	NS
Norplant/Implant	0.47 (0.98)	0.46 (0.96)	0.45 (1.04)	0.46 (0.98)	0.00	NS
Progestin only injection	2.87 (0.56)	3.00 (0.00)	2.45 (1.21)	2.87 (0.52)	4.33	*
Oral Pills	2.85 (0.56)	3.00 (0.00)	2.55 (1.04)	2.81 (0.59)	3.29	NS
Barrier Methods except condoms	0.42 (0.86)	0.78 (1.16)	0.45 (0.93)	0.17 (0.42)	6.23	*
Fertility awareness methods	1.67 (1.15)	1.95 (1.05)	2.27 (1.19)	1.39 (1.14)	4.45	**
Emergency Contraceptives	1.05 (1.32)	1.19 (1.43)	1.00 (1.34)	0.96 (1.26)	0.33	NS
Condoms	2.93 (0.35)	3.00 (0.00)	2.81 (0.60)	2.90 (0.40)	1.41	NS
Management of side effects	1.68 (1.08)	2.03 (0.87)	1.54 (1.04)	1.46 (1.18)	3.20	*

*p < 0.05; **p < 0.01; ***p < 0.001, NS-Not significant, SD = standard deviation, ANOVA- Analysis of variance.

Scores ranged from 0-3, P-value is from ANOVA test. PNFP- Private not for profit, PFP- private for profit

### Consent requirements to provide contraceptives to young people

More than a third of the providers (38%) requested consent from either a parent or spouse or both when a young person less than 18 years requested contraceptives. The proportion of providers requiring consent did not differ significantly by facility type. Fear of spousal or parental confrontation was the main reason why consent was deemed necessary. A majority of the providers (63.6%) in PNFP health facilities requested consent (n = 11). However, most of the providers said that consent was not necessary (Table 4).

**Table 4:** Consent requirement for contraceptive service provision to young people **<**18 years

	Health facility type				
	
Consent requirement for contraceptive service provision	*Total* *n = 102*	*Public* N = 37	*PNFP* N = 11	*PFP* N = 54	*Chi-sq* *p-value*
*Consent requirement*					
Consent from parent or spouse	39(38.2)	11(29.7)	7(63.6)	21(38.9)	NS
No consent required	63(61.8)	26(70.3)	4(36.4)	33(61.1)	
*Reason for consent (n = 39)*					
Fear of confrontation by spouse or parent	15 (14.7)	2 (5.4)	3 (27.3)	10(18.5)	
Discourage use by YP with no children	10(9.8)	5 (13.5)	2(18.2)	3(5.6)	
Fear that contraceptives are harmful	8 (7.8)	3 (8.1)	1 (9.1)	4 (7.4)	***
To scare young people from sex	6(5.9)	1 (2.7)	1 (9.1)	4 (7.4)	

*p < 0.05; **p < 0.01; ***p < 0.001, NS- Not significant, YP -young people, Note: number of respondents too small in some cells, Percentages within brackets. PNFP- Private not for profit, PFP- private for profit

### Provider perspectives on provision of contraceptives to young people

When asked for their views on provision of contraceptives to young people in an open ended question, most providers said that they were not prepared to provide contraceptives to young people. More than a third said that they would not provide contraceptives to those less than 18 years of age, unmarried, still in school, and those without children. Slightly less than one third of the providers believed that if young women used contraceptives early in life, they could have long-term side effects such as infertility. A fifth of the providers said that they would firmly discourage the use of the injectable method to young women due to perceived side effects. They would rather advise abstinence.

Some providers (14%) revealed that as parents, it was impossible to give contraceptives to young people because it was morally unacceptable. More than a fifth of providers said that they did not feel positive about giving contraceptives to young people, but considered that they had no choice. Those providers insisted on choosing the method they felt most appropriate for young people, usually condoms (17%). Nevertheless, these providers first used scare tactics to discourage young people from having sex. In addition, some providers felt too busy to attend to young people with contraceptive needs since they also had to deal with immunization and other services. Only a quarter of providers were comfortable giving contraceptives to sexually active young people, the other three quarters believed that contraceptives should not be provided to young people.

### Provider perspectives on health system obstacles to contraceptive service delivery for young people

Providers identified service delivery, provider-focused, and structural factors that influenced access to contraceptives for young people when asked in an open ended question for their opinion on health systems obstacles. Service delivery factors included the inconsistent and sporadic availability of contraceptive commodities (40%) and the lack of an appropriate method mix (12%) to meet young people's needs and preferences. Providers reported that some commodities like IUDs and implants often expired since no one asked for them. Providers highlighted poor infrastructure with limited space in which to offer both auditory and visual privacy during client consultations (39%), lack of appropriate equipment and educational materials (34%), and storage as other obstacles. Providers, especially in profit-oriented facilities, noted an absence of records or up-to-date registers.

Provider-focused factors identified included a lack of knowledge about contraceptives (45%), negative provider attitudes due to fears, myths, and health and safety concerns related to certain contraceptive methods (40%), and providers' denial or restrictions to provide all or some methods to young people (11%). Providers mentioned, for example, the risk that contraceptives would cause infertility. Structural factors cited were poor service quality, long waiting hours (32%), a limited number of qualified personnel, and high staff turnover (32%), as well as policy restrictions (17%). Constraints on funding for community sensitization, outreach, and support supervision were also considered barriers (34%).

### Provider perspectives on reasons why young people do not use contraceptives

In an open ended question, a large proportion of the providers were of the opinion that young peoples' lack of factual information and knowledge about contraceptives (72%) as well as fears of actual and perceived side effects (63%) were reasons why young people did not use contraceptives. Many providers also pointed out that young people have strong misconceptions about contraceptives (68%) and negative attitudes towards contraceptives (54%). Other reasons identified included long distances to clinics, the high cost of contraceptives, and unavailability of preferred contraceptive methods in facilities closest to home (62%). Providers said that community-related factors that limited contraceptive use among young people included opposition and disapproval by male partners (49%) and parents/peers (13%). Additional community-related factors reported by providers were cultural norms such as wishing for a big family (43%), religious beliefs (30%), and exchange of sex for money, and substance abuse (23%). Overall, providers viewed contraceptive service demand in their communities to be low, with young people constituting a minority of their clientele.

## Discussion

Providers' highlighted service delivery, provider, structural, and client-specific factors influencing young peoples' access to and use of contraceptives. The providers also cited inconsistent, sporadic availability, and poor method mix as limiting factors to methods of choice by clients in need of contraceptives. Long-acting methods that require fewer visits and have limited adherence or compliance problems were the least available methods. In addition, methods like hormone-releasing IUDs, combined hormonal patches, vaginal rings, diaphragms, and female condoms that would potentially benefit young women were not available at all. The availability of different contraceptive methods is known to influence contraceptive uptake. Research from low-income countries has shown that contraceptive use is higher when women have access to a variety of methods [24]. Increasing the availability of different methods as well as introducing new technologies might, thus, be a way to increase contraceptive use.

Policy guidelines, educational materials, and records of family planning services were limited in PFP facilities, which might indicate that their focus is on selling contraceptives rather than providing comprehensive contraceptive services. It also indicates poor collaboration between the public and private sector. However, PFP facilities were the majority and potentially important sources of contraceptives for young people. It was surprising that although the fertility awareness method was the most common method in PNFP facilities, there were no educational materials to promote it, further limiting prospects for adoption use of these methods.

Positive provider attitude is a key element in enhancing contraceptive use among young people. Our study indicates that the behavior of providers might influence young peoples' use or non-use of contraceptives. Many providers required parental or spousal consent and did not respect young people's choices, thereby denying them access to contraceptives. Our study further showed that contraceptive service providers imposed non-evidence-based age restrictions for certain contraceptive methods, a finding also cited in earlier studies in Tanzania and Ghana [25,26]. Parental or spousal consent was requested when young people below the Ugandan age of consent (18 years) sought contraceptive service, although the national guidelines do not have such a restriction [11]. The provider's health and safety concerns, especially regarding unmarried young women and women without children, resulted in hesitation in providing contraceptives. Providers mentioned, for example, the risk that contraceptives would cause infertility, which illustrates that providers have misconceptions just like their clients and the rest of the community, observations also noted in other studies [15,19]. Providers' restrictions and behavior might reflect their own personal attitudes and values [25,27], rather than evidence-based knowledge and national policy/guidelines.

In this study, providers assessed their services for young people as being unfriendly. Research studies have shown that when health care providers are friendly and welcoming, young people find it easier to discuss sensitive topics with them than with parents and teachers [19]. Providers also felt too busy with the provision of other services. This indicates that young peoples' needs, particularly contraceptive needs, are not prioritized and often considered unimportant or even time-consuming. The reason for this might be providers' views that young people should not use contraceptives.

The providers rated their own level of competence as rather low, particularly regarding comprehensive counseling, management of contraceptive side effects, provision of emergency contraception, fertility awareness and barrier methods, and long-acting methods such as IUDs and implants. The differences in competence by facility type were remarkable. PFP providers rated their competence to be low in providing most of the methods, indicating why many were not willing to communicate information about some of the methods. While the providers in drug shops/pharmacies are not expected to give IUDs and Implants, it is possible to sell these products and provide information or advice on these methods. In addition, they could refer the clients who need such methods. This is especially critical at points where such establishement may be an accessible source of contraceptive services. These findings demonstrate the need for training providers in all health care facilities irrespective of ownership. Lack of training was also reported as a barrier to family planning quality provision in another Ugandan study [28]. Our study showed that providers are not well-equipped to provide contraceptive services. Strategies to strengthen providers' knowledge and skills as well as changing restrictive attitudes are essential in order to improve access to contraceptive services and avert unwanted pregnancies.

Providers believed and emphasized that client-related factors such as myths, limited knowledge about contraceptives, and poor understanding of available services prohibited young people from using contraceptives. Furthermore, providers referred to community perspectives and norms that inhibited contraceptive use, and also maintained that there was opposition and disapproval from parents and spouses. Religious restrictions were said to be an important barrier to contraceptive use, particularly at PNFP health facilities.

Cost in terms of transport and time and the cost of contraceptives were also considered an important constraint to young people's access to contraceptive service, not only in private facilities, but also in public health care facilities where contraceptives are free of charge. This illustrates how poverty influences young peoples' access to contraceptive services, which has also been highlighted in client-based studies [12,15]. It is worth noting that contraceptive failure or nonuse is probably more costly, particularly to women who have no access to safe abortions [8,27].

While there were a number of hindrances to contraceptive access and use, the study showed some positive opportunities regarding young peoples' access to contraceptives. Many providers had, for example, recently provided contraceptives to young people, and a large proportion of providers expressed competence to provide pills, injections and male condoms and, to a limited extent, also fertility awareness methods. Another positive aspect was that two thirds of providers did not impose any consent requirements. Also, clients who were married and those with children had access to contraceptives, which is a positive finding for prospective contraceptive users. In addition, signposts about family planning were available at all public and PNFP health care facilities. Some PFP facilities did not provide contraceptive services and were, thus, not included in our study. These facilities are potential sites for future contraceptive service delivery, indicating prospects for future improved access and use.

This study brings out several program and policy implications. Firstly, gaps in providers' knowledge and competence need to be urgently addressed in order to scale up access to contraceptives for young people. Secondly, nurses and midwives who comprise the majority of providers should be trained by government and encouraged to give long-acting methods at all levels of the decentralized health sector in order to improve access. Thirdly, health systems need to be strengthened financially to ensure commodity security and other logistics. Wide dissemination of policy guidelines in public and private sectors beyond facility based providers is essential. Fourthly, re-orienting health care system to integrate contraceptive services in outpatient departments should be introduced. Finally, adoption of low-cost, highly effective, and longer-acting methods, such as implants, would reduce financial burdens, thus benefitting young people who tend not to be endowed with enough resources.

Our study was limited by the number of health facilities and the use of data that relied on contraceptive service provider perceptions and reports. The selection of providers might have had an impact on the study results. The study did not observe provider-client interactions or interview clients; thus, the effect of provider attitudes and behavior on young peoples' contraceptive use was not ascertained. We focused on providers' views of young people in general, discounting sex, so we cannot conclude if providers' views were related to girls or boys or both.

## Conclusion

Our results indicate that provider, client, and health system factors are constraints on the use and provision of contraceptives for young people. The majority of the providers had the view that young people should not use contraceptives. There was limited access to long-acting methods, sporadic stocks, and a shortage of competent personnel. Most providers were not competent enough to provide long-acting methods, emergency contraceptives and barrier methods apart from condoms. Provider behaviors such as imposing non-evidence-based requirements, refusal, and restrictions probably influence the service young people receive negatively and further constrain access to and use of contraceptives. Unmarried women in Uganda are given the message by everyone that they have no business using contraceptives if they are not married and not planning a family. The low and inconsistent use of contraceptives among young people might be attributable a shortfall in the health care delivery system. Contraceptive use prospects for young people depend on technical competence, provider behavior, and health system improvements in addition to addressing demand side factors.

## List of Abbreviations

FAM: Fertility Awareness Methods; HSD: Health Sub District; IUD: Intra-Uterine Device; PFP: Private for profit; PNFP: Private not for profit; YP: Young people.

## Competing interests

The authors declare that they have no competing interests.

## Authors' contributions

GN planned the study, conducted the field-work, coded the open-ended questions, and had the main responsibility for analyzing data and for writing the manuscript. EF and FM contributed to the design and the development of research tools, and supervised the writing of the manuscript. JB contributed to writing of the manuscript. NT contributed to the analysis and statistics components. All authors read and approved the final manuscript.

## Pre-publication history

The pre-publication history for this paper can be accessed here:

http://www.biomedcentral.com/1472-6963/11/220/prepub
